# Renal Metastasis from Extrahepatic Cholangiocarcinoma Mimicking Renal Infarction: A Case Report and Literature Review

**DOI:** 10.3390/diagnostics16142207

**Published:** 2026-07-15

**Authors:** Mirela-Georgiana Perne, Olga Hilda Orășan, Cezara Andreea Gerdanovics, Mircea Vasile Milaciu, Călin Vasile Vlad, Ioan Șimon, Răzvan Togănel, Nicolae Voicu Rednic, Iuliana Georgiana Țifui, Cristina Elena Cornea, Nicoleta Valentina Leach, Codruța Claudia Gherman Lencu, Anamaria Vlăduța Tomoiagă, Vasile Negrean, Teodora Gabriela Alexescu

**Affiliations:** 1 Department of Internal Medicine, 4th Medical Discipline, “Iuliu Hațieganu” University of Medicine and Pharmacy, Republicii Street, No. 18, 400015 Cluj-Napoca, Romaniavladvasilecalincfcluj@gmail.com (C.V.V.); vladutat@yahoo.com (A.V.T.); vasile.negrean@umfcluj.ro (V.N.); teodora.alexescu@umfcluj.ro (T.G.A.); 2 4th Surgical Department, “Iuliu Hațieganu” University of Medicine and Pharmacy, Republicii Street, No. 18, 400015 Cluj-Napoca, Romania; 3 Regional Institute of Gastroenterology and Hepatology “Prof. Dr. Octavian Fodor”, 400394 Cluj-Napoca, Romania; 4 Municipal Hospital Aiud, 515200 Aiud, Romania; cristina.cornea99@gmail.com; 5 Department 2, Faculty of Nursing and Health Sciences, “Iuliu Hațieganu” University of Medicine and Pharmacy, Republicii Street, No. 18, 400015 Cluj-Napoca, Romania; 6 Department 1, Fundamental Discipline, Endocrinology, “Iuliu Hațieganu” University of Medicine and Pharmacy, Victor Babeș Street, No. 8, 400347 Cluj-Napoca, Romania; codruta_lmc@yahoo.com

**Keywords:** cholangiocarcinoma, extrahepatic cholangiocarcinoma, renal metastasis, renal infarction, contrast-enhanced ultrasound, immunohistochemistry

## Abstract

**Background:** Cholangiocarcinoma (CCA) is a rare and aggressive malignancy of the biliary tree. Renal metastasis represents an exceptionally rare event, reported in fewer than 0.5% of cases. Its clinical and imaging presentation may closely mimic benign renal conditions, particularly renal infarction, posing a significant diagnostic challenge. **Case Presentation:** We report the case of a 72-year-old man who initially presented with intense right-sided flank pain without urinary symptoms. Combined imaging findings, including absent Doppler signal on ultrasound and absent contrast uptake on contrast-enhanced ultrasound (CEUS) in two-thirds of the right renal parenchyma, led to an initial diagnosis of right renal infarction. Seven months later, re-evaluation prompted by macroscopic haematuria, significant weight loss, cholestatic syndrome, and markedly elevated CA 19-9 revealed an invasive renal tumour mass associated with biliary ductal dilatation. Renal biopsy with immunohistochemical analysis (CK7+, PAX8−, c-kit weakly positive) confirmed renal metastasis from extrahepatic cholangiocarcinoma. The patient’s clinical course was complicated by acute cholangitis, Clostridioides difficile enterocolitis, and upper gastrointestinal haemorrhage, requiring endoscopic retrograde cholangiopancreatography (ERCP) with biliary stenting, endoscopic ultrasound (EUS)-guided hepaticogastrostomy, and endoscopic haemostasis. **Conclusions:** A systematic review of the published literature identified only two previous dedicated case reports of renal metastasis from CCA, making this, to our knowledge, the third such case and the first involving an extrahepatic primary tumour presenting through its renal metastasis. This case highlights the diagnostic pitfall of mistaking hypovascular renal metastasis for renal infarction, the indispensable role of immunohistochemistry, and the importance of early percutaneous biopsy in avascular renal lesions without a confirmed thromboembolic aetiology.

## 1. Introduction

Cholangiocarcinoma (CCA) accounts for approximately 3% of all gastrointestinal malignancies and is the second most common primary hepatic malignancy after hepatocellular carcinoma [1,2]. It predominantly affects men, with a mean age at diagnosis of 50–70 years [1,2,3,4,5,6,7,8,9,10,11,12,13,14,15]. Extrahepatic CCA (eCCA)—comprising perihilar and distal subtypes—is globally increasing in incidence and is characterised by late presentation, high recurrence rates, and a 5-year overall survival that rarely exceeds 30% even after curative resection [16].

Risk factors include primary sclerosing cholangitis, cholelithiasis, choledochal cysts, liver cirrhosis, chronic viral hepatitis B or C, hepatic trematodes (Opisthorchis viverrini, Clonorchis sinensis), and genetic predispositions (Lynch syndrome, cystic fibrosis) [5].

CCA is classified anatomically into intrahepatic (iCCA) and extrahepatic (eCCA) subtypes. eCCA is further divided into perihilar and distal forms. Perihilar CCA is subclassified by the Bismuth-Corlette system into five types: type 1, confined to the common hepatic duct; type 2, involving the hepatic bifurcation extending into both hepatic ducts without reaching secondary branches; type 3a/3b, extending into the right or left hepatic duct respectively; and type 4, involving both ducts bilaterally [1,6].

Clinical presentation is dominated by features of biliary obstruction features: jaundice, pruritus, clay-coloured stools, and dark urine, accompanied by abdominal pain, weight loss, fever, malaise, and night sweats [3,7]. Elevated CA 19-9 and CEA, alongside cholestatic liver biochemistry, support the diagnosis [8]. Imaging (ultrasound, CT, MRI) and endoscopic evaluation (ERCP, EUS) are the mainstays of staging and tissue acquisition [1,9,10].

The most common metastatic sites for eCCA are the liver, distant lymph nodes, and lungs [11,12,13,14,15,16,17]. Renal involvement is exceedingly rare, reported in fewer than 0.5% of cases [11,12,13]. We present a unique case of eCCA in which the renal metastasis constituted the initial clinical manifestation and closely mimicked renal infarction on imaging.

## 2. Case Presentation

### 2.1. First Admission (September 2024)

A 72-year-old male from a rural area, with a history of grade III essential hypertension, hypercholesterolaemia, and seronegative rheumatoid arthritis, presented in September 2024 with severe right-sided flank pain radiating to the right iliac fossa, without urinary symptoms. Initial evaluation at a local emergency department, including abdominal ultrasound, was unremarkable; the presentation was attributed to lumbar syndrome with sciatica.

Following progressive symptom worsening, the patient was referred to Cluj-Napoca CF Clinical Hospital. Physical examination revealed moderately impaired general condition, mild tenderness in the right hypochondrium and iliac fossa, and a positive right-sided Giordano sign. Laboratory findings included an inflammatory syndrome (elevated CRP, ESR), mild normochromic normocytic anaemia (Hb 11.9 g/dL), mild azotaemia, and microscopic haematuria.

Abdominal ultrasound showed a hyperechoic hepatic lesion in segment IV (suggestive of haemangioma) and an enlarged right kidney with absent Doppler signal in approximately two-thirds of the parenchyma (Figure 1).

Contrast-enhanced ultrasound (CEUS) confirmed absent contrast uptake in extensive areas of the right kidney (Figure 2).

Contrast-enhanced CT of the abdomen and pelvis demonstrated a hypodense hepatic lesion (Figure 3), avascular parenchyma in the majority of the right kidney (Figure 4), and absent opacification in the urographic phase—findings raising suspicion for renal infarction, tumour or pyelonephritis (Figure 5).

Following urological and surgical consultation, the working diagnosis of right renal infarction was established. Thromboembolic workup (atrial fibrillation screen, thrombophilia panel, hypercoagulable state evaluation) was performed without identifying a definitive embolic source. Conservative anticoagulation therapy was initiated; revascularisation was not performed.

### 2.2. Re-Admission (April 2025)

Seven months later, the patient presented with persistent right flank pain, new-onset macroscopic haematuria, and significant unintentional weight loss. Laboratory evaluation revealed an accentuated inflammatory syndrome, leucocytosis with neutrophilia, cholestatic syndrome (markedly elevated GGT and ALP), worsening anaemia, and a significantly elevated CA 19-9.

Repeat CT imaging demonstrated an invasive renal tumour mass with invasion of the right renal pedicle and encasement of the right renal artery, associated with hypodense hepatic lesions and intrahepatic bile duct dilatation (Figure 6, Figure 7 and Figure 8).

Hepatic CEUS (Figure 9 and Figure 10) and CT-guided renal biopsy with immunohistochemical analysis (Figure 11) revealed an epithelial tumour proliferation negative for primary renal tumour markers but consistent with a metastasis of biliary–pancreatic, gastro-oesophageal, pulmonary, breast or head-and-neck origin.

A systematic primary site search—upper GI endoscopy, testicular ultrasound, ENT consultation, and chest radiograph—was unremarkable. The immunohistochemical profile comprised CK7-positive (Figure 12), c-kit weakly positive (Figure 13) and PAX8-negative (Figure 14). The absence of PAX8 excluded a primary renal origin; CK7 positivity directed diagnosis toward a biliary or pancreatobiliary primary [18,19,20,21,22,23]. The tumour was staged as cT4 cN1 cM1 (stage IV eCCA). Curative resection was not feasible.

### 2.3. Clinical Course and Complications

The clinical course was complicated by cholestatic liver disease secondary to tumour-related biliary obstruction. Infectious, viral, parasitic, toxic, and drug-induced hepatic aetiologies were systematically excluded.

Endoscopic biliary decompression via ERCP with partial common bile duct stenting was performed (Figure 15). Post-ERCP acute cholangitis was treated empirically with broad-spectrum antibiotics, subsequently complicated by Clostridioides difficile-associated enterocolitis.

Due to incomplete biliary drainage, EUS-guided hepaticogastrostomy (EUS-HGS) was performed, achieving biliary drainage into the stomach (Figure 16).

The subsequent course was marked by haemodynamic instability and melaena with progressive haemoglobin decline. Angio-CT demonstrated hepatogastric stent positioning, intragastric haemorrhagic content, malignant hepatic lesions with vascular invasion, thrombosis of the right portal vein branch and right hepatic artery, and right intrahepatic bile duct dilatation. Upper GI endoscopy identified an active subcardial ulcer; haemostasis was achieved with metallic clips.

Despite ongoing melaena and haemoglobin decline, haemodynamic stability was maintained. Repeat EUS confirmed patent hepaticogastrostomy with active bile flow, absence of active gastric bleeding, and an adherent papillary clot; the plastic biliary stent was extracted and haemostasis completed.

At discharge: reduced performance status (ECOG 3), partially improved cholestatic syndrome, and partially corrected anaemia. A high-protein diet, hepatoprotective agents, and analgesic therapy were recommended. Oncological multidisciplinary board review was completed; systemic chemotherapy (gemcitabine plus cisplatin) was discussed but deferred pending clinical optimisation given the patient’s ECOG 3 status. Molecular tumour profiling was not performed due to rapid clinical deterioration.

## 3. Discussion

### 3.1. Renal Metastasis from Cholangiocarcinoma: A Systematic Review of Reported Cases

A PubMed search using the terms “cholangiocarcinoma,” “renal metastasis,” and “metastasis to the kidney” identifies only two dedicated case reports and two incidental cases within surgical series on recurrent iCCA [11,12], confirming the extreme rarity of this entity.

Choi et al. (2012) reported the first case of renal metastasis from CCA—a 64-year-old male with iCCA, four months after hepatic resection and cholecystectomy. CT suggested a renal abscess; left nephrectomy revealed a non-encapsulated tumour with squamous differentiation confirmed as CCA metastasis. Pulmonary metastases followed, and the patient died approximately 14 months post-nephrectomy [12].

Matsumoto et al. (2020) described a 72-year-old male with iCCA (pT3N0M0, 2014) who developed synchronous hepatic recurrence and a left renal mass in 2017. PET-CT showed uptake in both lesions; simultaneous partial nephrectomy and hepatic resection were performed. IHC (CK7+, CK19+, CK20−, PAX8−) confirmed renal metastasis from iCCA. Bone and pulmonary metastases appeared four months later; the patient commenced gemcitabine/cisplatin and remained alive at 30 months [11].

Two further incidental cases appear in surgical series: Kamphues et al. included one patient who underwent right nephrectomy combined with repeat hepatic resection, and Yoh et al. reported one resected renal metastasis in a 15-patient series—both demonstrating potential benefit of surgical resection in selected cases [11].

The present case is distinguished by three features absent from prior reports: (1) the renal metastasis constituted the initial clinical presentation of an occult eCCA, rather than a recurrence following a known primary; (2) the lesion fulfilled all imaging criteria for renal infarction, a diagnostic pitfall not previously described in this context; and (3) the primary was extrahepatic rather than intrahepatic.

A comparative summary is presented in Table 1.

### 3.2. Epidemiology and Metastatic Patterns of Extrahepatic Cholangiocarcinoma

eCCA is a globally increasing malignancy; most patients present with locally advanced or metastatic disease, and optimal survival remains constrained by high recurrence risk [16]. A SEER-based cohort analysis of 4061 patients with extrahepatic bile duct cancer by Wang et al. identified the liver and distant lymph nodes as the most frequent single-organ metastatic sites, with the lung predominating in bi-organ patterns [17]. Renal involvement was not identified as a distinct metastatic pattern—consistent with its sub-0.5% incidence—reinforcing the exceptional nature of our case.

For iCCA, Hahn et al. (2020) identified the lung (n = 105), peritoneum (n = 81), and bone (n = 50) as the most frequent metastatic sites in 370 patients over 20 years; renal metastases were not categorised as a discrete entity [13].

A parallel is offered by a 2025 case report (Jiang et al.) of iCCA presenting through rectal metastasis, where intestinal symptoms preceded identification of the hepatic primary [18]. As in our case, the index lesion was the metastasis, and IHC of the metastatic biopsy was the pivotal diagnostic step—underscoring the theme that CCA may first manifest at an atypical metastatic site.

### 3.3. Differential Diagnosis: Renal Infarction Versus Hypovascular Renal Metastasis

Renal infarction is rare; in a retrospective analysis of 186 consecutive cases, Faucon et al. found atrial fibrillation to be the leading aetiology (56%) [14]. In the absence of a confirmed embolic source—as in our patient—clinical evolution diverging from the expected pattern of progressive parenchymal scarring should prompt diagnostic reassessment. In such cases, careful review of CT imaging by an experienced uro-radiologist within a multidisciplinary setting is warranted, particularly when the clinical evaluation is not fully consistent with the initial diagnosis of renal infarction. Once pyelonephritis has been excluded on clinical and laboratory grounds, and renal infarction becomes less likely due to the absence of a clear thromboembolic source or an atypical clinical course, an underlying renal neoplasm or renal metastasis should be actively reconsidered. In this scenario, early involvement of a uro-oncology multidisciplinary team, including a urologist, radiologist, oncologist and radiotherapist, may help integrate radiological, clinical and laboratory data and guide the decision regarding whether renal biopsy should be performed or temporarily deferred.

A series of 35 cases of renal metastases from non-renal primaries (Lian et al., 2023) found that CT correctly diagnosed renal metastasis in only 45.5% of cases, while renal biopsy achieved a diagnostic yield of 93.8% [19]. These data directly support the diagnostic strategy in our case.

On imaging, both renal infarction and hypovascular metastases present as avascular, hypodense areas on CT with absent CEUS enhancement. In CT evaluation of renal lesions, contrast enhancement should be assessed quantitatively by comparing attenuation values on unenhanced and post-contrast images. An increase greater than 20 Hounsfield Units is generally considered to represent true enhancement and supports the diagnosis of a solid renal mass rather than a non-enhancing infarcted or cystic lesion.

The differential diagnosis of a hypovascular renal lesion with irregular or infiltrative borders should also include rare primary renal or urothelial malignancies. As summarised by Bazzocchi et al., renal masses include a broad spectrum of entities, including RCC subtypes, transitional/urothelial carcinoma, metastases and lymphoma. Heterogeneous renal masses with attenuation between 20 and 70 HU on unenhanced CT are considered indeterminate and require multiphasic CT or MRI evaluation, while true enhancement is generally defined by an attenuation increase greater than 20 HU. Upper urinary tract transitional/urothelial carcinoma, although rare and accounting for less than 5% of renal tumours, usually arises from the renal pelvis and is best assessed on the urographic phase, where filling defects, mural thickening, obstructed calyces or hydronephrosis may be detected. Renal lymphoma should also be considered, as the kidney is the most commonly involved genitourinary organ in extranodal lymphoma, although primary renal lymphoma is rare. Since renal metastases may also show ill-defined margins and low-level enhancement, biopsy is essential when imaging and clinical data remain inconclusive [20]. Distinguishing features favour infarction when lesions are sharply demarcated and wedge-shaped with a cortical rim sign, associated with identifiable thromboembolic risk factors. Metastases tend to have irregular margins, extracapsular extension, or pedicle invasion—features that only became apparent on follow-up imaging in our patient [11,12,14,15,19].

The AUA guidelines on renal mass evaluation recommend biopsy specifically when metastasis from another primary is suspected [21]. In oncological patients with avascular renal lesions and no confirmed embolic source, early biopsy should be considered—a lesson reinforced by the seven-month diagnostic delay in our case.

### 3.4. Immunohistochemical Diagnosis

Immunohistochemistry was essential in this case. PAX8 is highly sensitive for renal, thyroid, ovarian, and endometrial carcinomas; its negativity combined with diffuse CK7 positivity directed diagnosis toward a biliary or pancreatobiliary primary [22,23]. A 2023 study by Mocan et al. from the same academic centre (Iuliu Hațieganu University, Cluj-Napoca) validated a CK7 sensitivity of 93.7% for iCCA on needle biopsy, and found CK7 positivity to be associated with a trend toward improved overall survival [22]. Choi et al. (2024) further delineate the panel: clear cell RCC is PAX8+/CAIX+/RCC-Ma+, while CCA is typically CK7+/CEA+/MUC2+/MUC5AC+ [23].

A critical gap in the present case—and a limitation shared across all published cases of CCA renal metastasis—is the absence of molecular tumour profiling. Comprehensive genomic profiling (CGP) identifying actionable alterations (FGFR2 fusions, IDH1 mutations, ERBB2 amplification) could have therapeutic implications even in the palliative setting. This should be considered a standard-of-care recommendation in future similar cases.

### 3.5. Palliative Biliary Drainage

Biliary obstruction is a cardinal complication of advanced CCA. In our patient, partial ERCP stenting required escalation to EUS-HGS, a validated rescue procedure [24]. A 2024 comprehensive review by Scholl et al. confirms technical success rates exceeding 90% for EUS-HGS in experienced centres [24]. Schoch et al. (2022) [25] specifically assessed EUS-HGS in perihilar CCA, reporting a median overall survival of 91 days (range 31–263), with 30-day and 3-month mortality of 23.5% and 47.1% respectively—reflecting disease burden rather than procedural failure, as 64.3% of deaths were attributed to disease progression [24]. Independent predictors of complications included Bismuth type II–IV CCA, hepatic metastases, ascites, and bilirubin >30 mg/dL—all features present in our patient [25].

The cascade of complications—cholangitis, Clostridioides difficile enterocolitis, gastrointestinal haemorrhage—illustrates the cumulative iatrogenic morbidity vulnerability of patients with advanced biliary malignancy and underscores the need for early, comprehensive multidisciplinary planning.

### 3.6. Systemic Therapy and Future Directions

The therapeutic landscape for advanced CCA has substantially evolved. The TOPAZ-1 and KEYNOTE-966 trials established gemcitabine/cisplatin plus durvalumab or pembrolizumab as the new first-line standard for advanced biliary tract cancer, demonstrating consistent overall survival benefit versus chemotherapy alone [26]. In our patient, systemic therapy was deferred due to ECOG 3 status; however, these regimens should be considered following clinical stabilisation.

Beyond first-line chemo-immunotherapy, CGP has identified multiple actionable targets: FGFR2 fusions/rearrangements (pemigatinib, futibatinib—FDA-approved second-line), IDH1 mutations (ivosidenib—FDA/EMA-approved), HER2 amplifications, BRAF V600E mutations, and NTRK/RET fusions [26,27]. Routine CGP at diagnosis, even in stage IV eCCA with atypical metastatic presentations, is strongly supported by current evidence.

The absence of molecular profiling, genomic data, and treatment response information in all published cases of CCA renal metastasis represents a significant gap. Prospective international case registries would be required to generate meaningful data on this entity.

## 4. Limitations

This report carries several limitations inherent to its design and clinical context that must be acknowledged.

**Single-case design.** As a case report, the findings cannot be generalised. No conclusions regarding incidence, prognosis, or treatment efficacy in this specific metastatic pattern can be drawn from a single observation. The extremely limited number of published cases (n = 3 including ours) precludes any systematic analysis of risk factors or outcomes.

**Absence of molecular tumour profiling.** Comprehensive genomic profiling (CGP) was not performed in this patient due to rapid clinical deterioration and ECOG 3 performance status at the time of histological diagnosis. As a consequence, potentially actionable alterations (FGFR2 rearrangements, IDH1 mutations, ERBB2 amplification, microsatellite instability status) remain unknown. This limits the oncological discussion to theoretical considerations and precludes reporting on targeted therapy eligibility—a significant limitation given the current precision oncology landscape.

**Diagnosis by exclusion.** The primary tumour was never directly biopsied or resected. The diagnosis of extrahepatic CCA was established through immunohistochemical characterisation of the renal metastasis and systematic exclusion of other primary sites. Although the initial segment IV hepatic lesion, interpreted at that time as suggestive of haemangioma, could not be retrospectively confirmed as the primary tumour, the IHC profile of the renal metastasis (CK7+, PAX8−), together with the clinical and imaging context of biliary obstruction, intrahepatic bile duct dilatation and elevated CA 19-9, is strongly consistent with a biliary primary. Nevertheless, absolute anatomical confirmation is lacking.

**Incomplete follow-up data.** The patient was discharged with ECOG 3 status and referred for oncological follow-up. Long-term survival data, response to any subsequent systemic therapy, and post-discharge quality-of-life information are not available at the time of this report.

**Absence of cross-sectional imaging of the primary tumour at initial presentation.** In retrospect, the hepatic lesion identified on initial ultrasound as a ‘haemangioma’ in segment IV warrants critical reassessment. While CEUS and CT findings at the first admission did not characterise it as malignant, the possibility that this represented the primary tumour cannot be excluded. This retrospective ambiguity is an inherent limitation of the diagnostic sequence.

**Ethics committee approval.** This manuscript is a case report based on data obtained during routine clinical care and follow-up. No additional diagnostic or therapeutic procedures were performed for research purposes. Written informed consent for publication of the anonymized clinical information and imaging data was obtained from the patient. Institutional ethics approval was obtained specifically for the publication of this case report from the Ethics Committee of the Cluj-Napoca CF Clinical Hospital, approval/minutes no. 10/04.06.2026

## 5. Conclusions

This case represents a rare and diagnostically challenging presentation of extrahepatic cholangiocarcinoma, in which renal metastasis constituted the initial clinical manifestation and closely mimicked renal infarction on imaging. Correct diagnosis required multidisciplinary re-evaluation triggered by clinical alarm signs, supported by sequential imaging, and confirmed by renal biopsy with immunohistochemical analysis.

The systematic literature review confirms that renal metastasis from CCA is exceedingly rare (<0.5%), with only two prior dedicated case reports documented. The present case is, to our knowledge, the first involving an extrahepatic primary and the first in which renal metastasis was the presenting manifestation. When the clinical course is inconsistent with renal infarction and pyelonephritis has been excluded, early multidisciplinary reassessment with uro-radiology and uro-oncology input is warranted to reconsider renal neoplasm or metastasis and to guide the timing of renal biopsy.

Four key clinical lessons emerge: (1) an avascular renal lesion without a confirmed thromboembolic source in a patient with systemic signs of malignancy warrants early biopsy rather than watchful waiting; (2) a panel of IHC markers—particularly PAX8 and CK7—is essential and sufficient to distinguish primary renal carcinoma from biliary metastasis; (3) the management of advanced CCA with biliary obstruction requires anticipatory multidisciplinary planning to limit cumulative iatrogenic morbidity; and (4) molecular tumour profiling should be pursued at diagnosis in all stage IV CCA patients, irrespective of metastatic site, to enable personalised therapy.

## Figures and Tables

**Figure 1:**
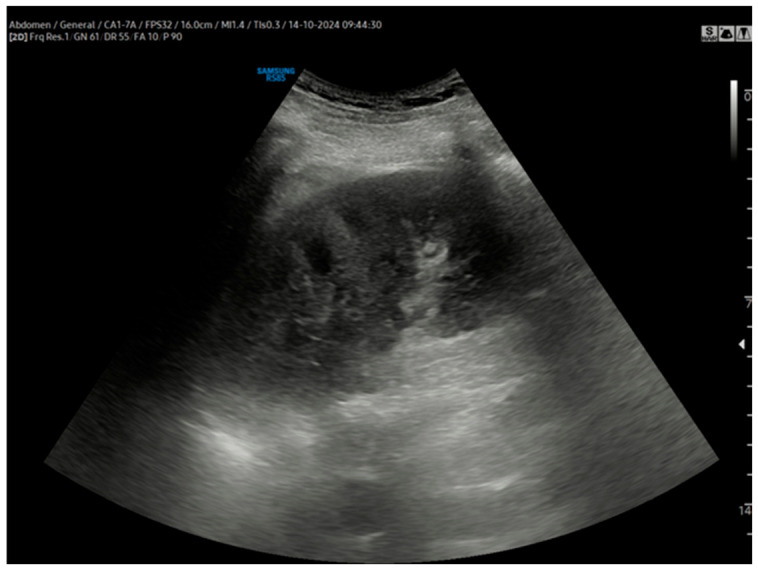
Grayscale and Doppler ultrasound of the right kidney showing renal enlargement, with absent Doppler vascular signal in approximately two-thirds of the renal parenchyma, predominantly involving the mid and lower renal regions.

**Figure 2:**
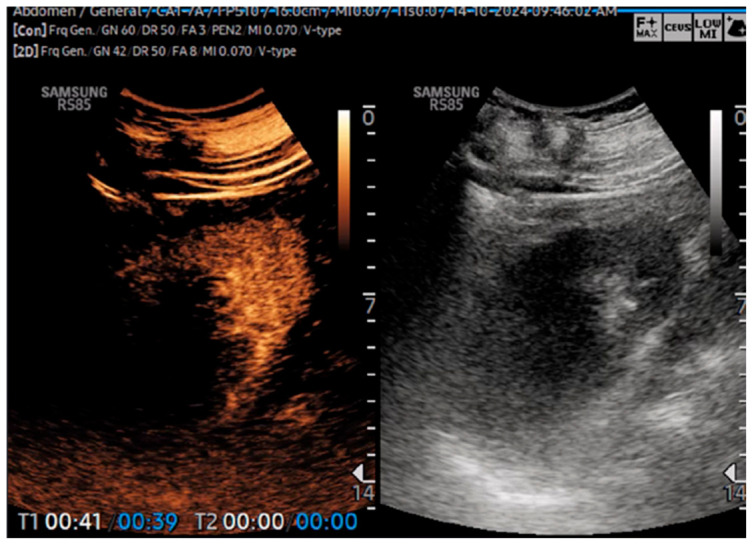
Contrast-enhanced ultrasound (CEUS) of the right kidney demonstrating markedly reduced to absent contrast enhancement in approximately two-thirds of the renal parenchyma, indicating an extensive hypovascular area and initially supporting the suspicion of renal infarction.

**Figure 3:**
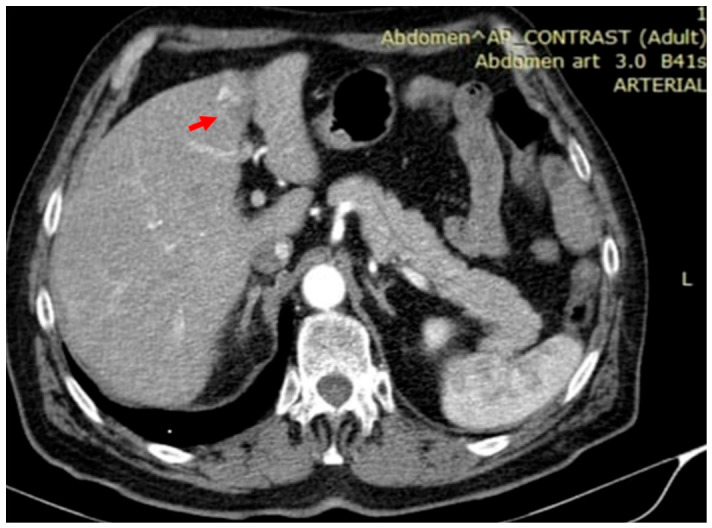
Arterial-phase contrast-enhanced abdominal and pelvic CT showing a hepatic lesion with peripheral discontinuous nodular enhancement, indicated by the arrow. The lesion was hypodense on the unenhanced phase and was initially interpreted as suggestive of hepatic haemangioma.

**Figure 4:**
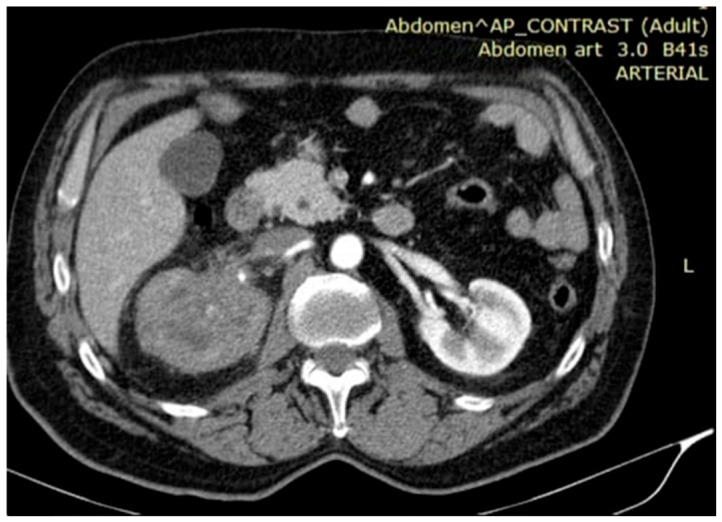
Contrast-enhanced abdominal and pelvic CT demonstrating extensive avascular parenchymal areas involving most of the right kidney, supporting the initial suspicion of renal ischemia.

**Figure 5:**
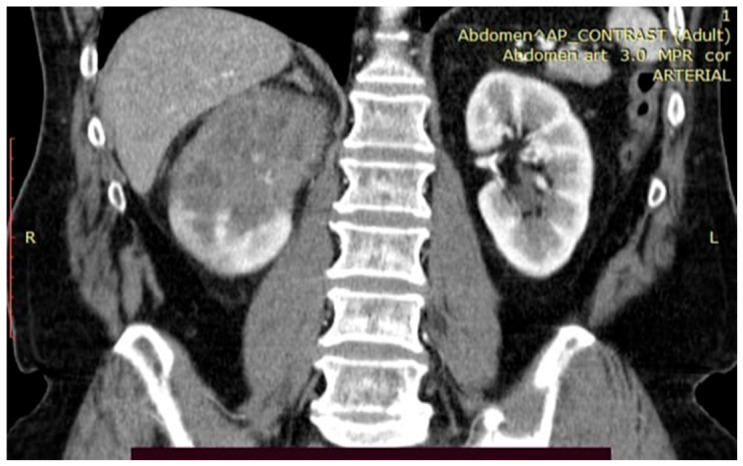
Urographic-phase contrast-enhanced abdominal and pelvic CT showing absent opacification of a large portion of the right kidney. In the initial clinical context, the differential diagnosis included renal infarction, renal tumour and pyelonephritis.

**Figure 6:**
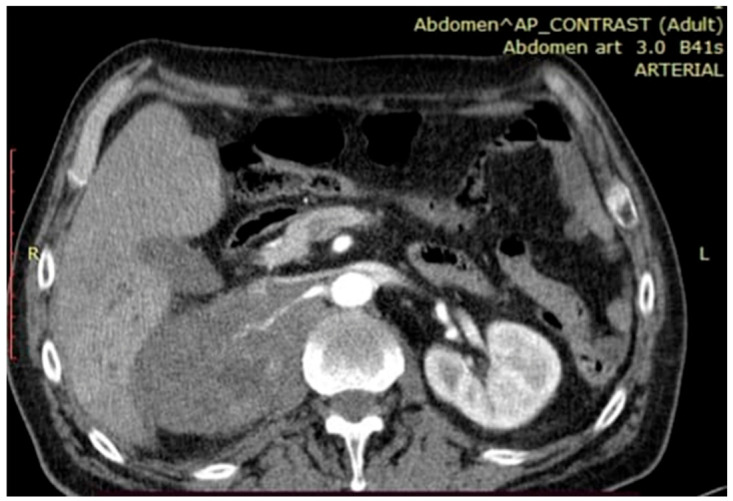
Repeat contrast-enhanced CT showing tumour invasion of the right renal pedicle with encasement of the right renal artery.

**Figure 7:**
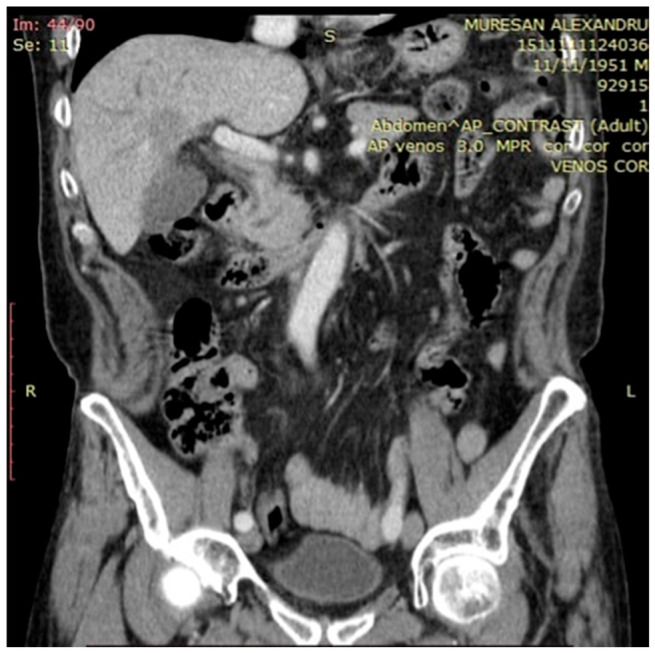
Repeat contrast-enhanced CT demonstrating hypodense hepatic lesions associated with intrahepatic bile duct dilatation.

**Figure 8:**
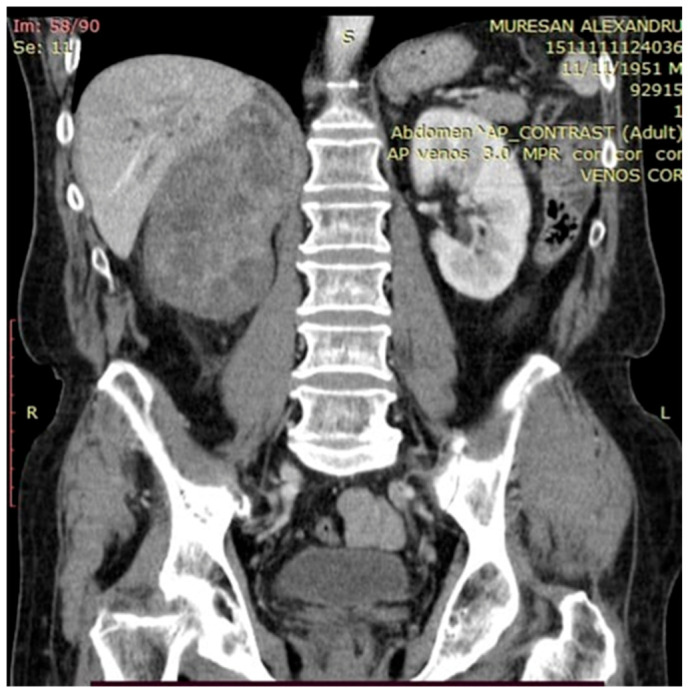
Repeat contrast-enhanced CT showing an invasive right renal tumour mass replacing and infiltrating the renal parenchyma.

**Figure 9:**
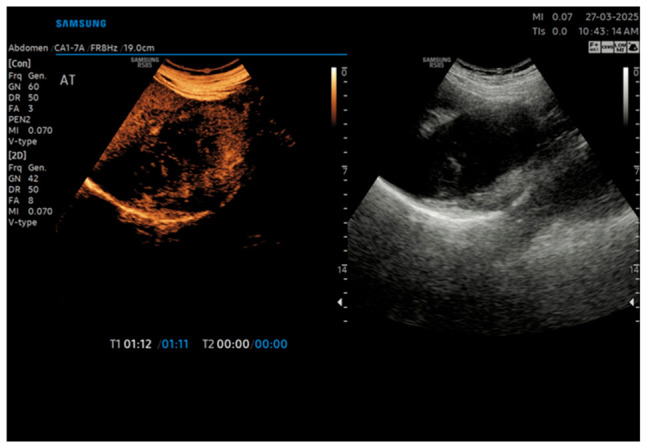
Contrast-enhanced ultrasound (CEUS) of the right kidney showing renal enlargement with absent contrast enhancement in extensive areas of the renal parenchyma.

**Figure 10:**
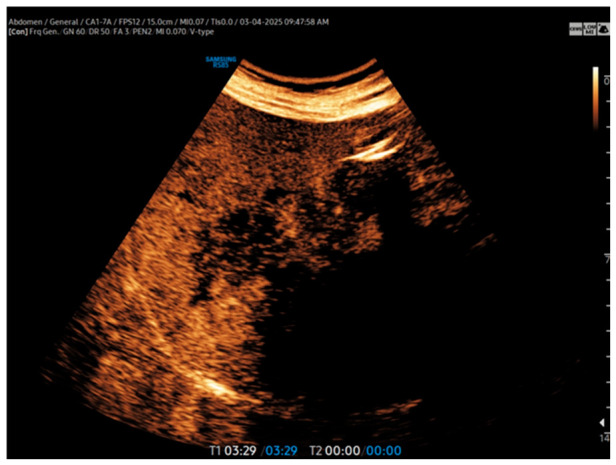
Hepatic contrast-enhanced ultrasound (CEUS) demonstrating heterogeneous liver parenchyma with multiple hypoenhancing areas, suspicious for malignant hepatic involvement.

**Figure 11:**
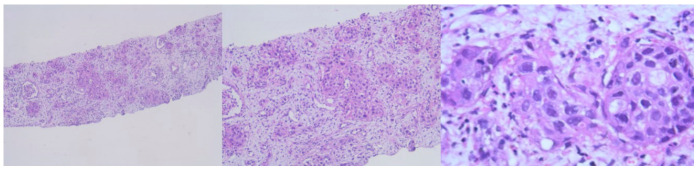
Microscopic examination of the CT-guided renal biopsy specimen showing epithelial tumour proliferation infiltrating the renal tissue.

**Figure 12:**
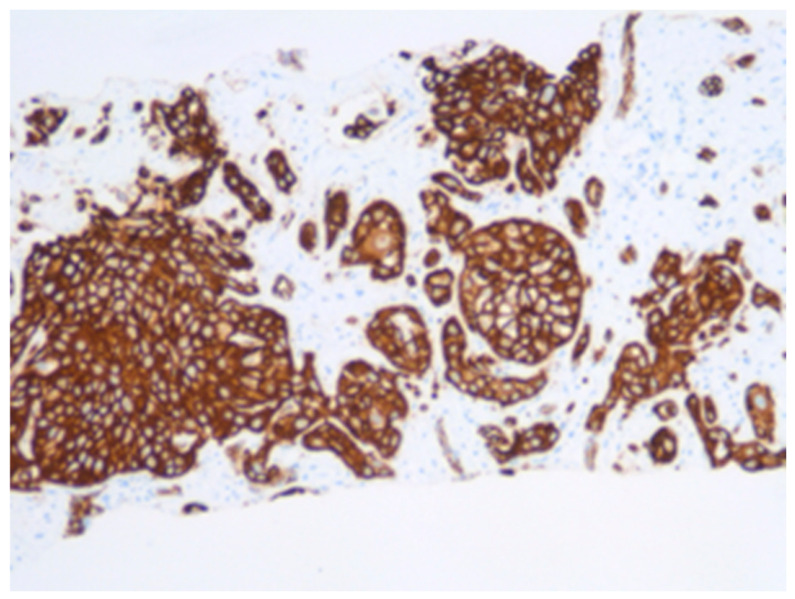
Immunohistochemical staining showing CK7 positivity in the tumour cells.

**Figure 13:**
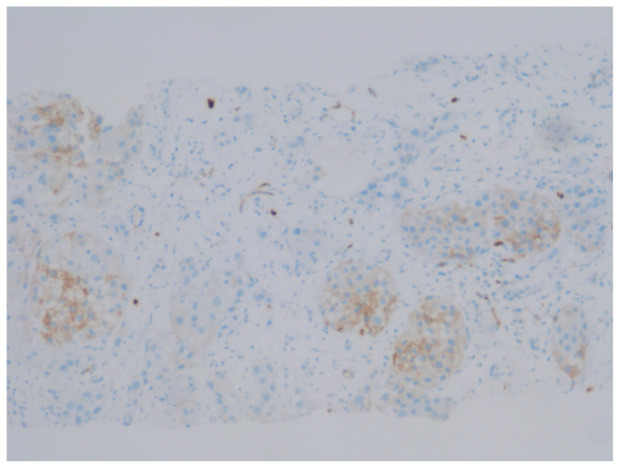
Immunohistochemical staining showing weak c-kit positivity in the tumour cells.

**Figure 14:**
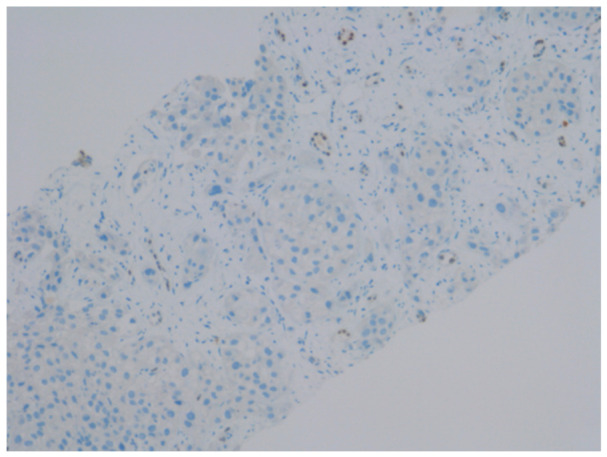
Immunohistochemical staining showing negative PAX8 expression in the tumour cells, arguing against a primary renal epithelial tumour.

**Figure 15:**
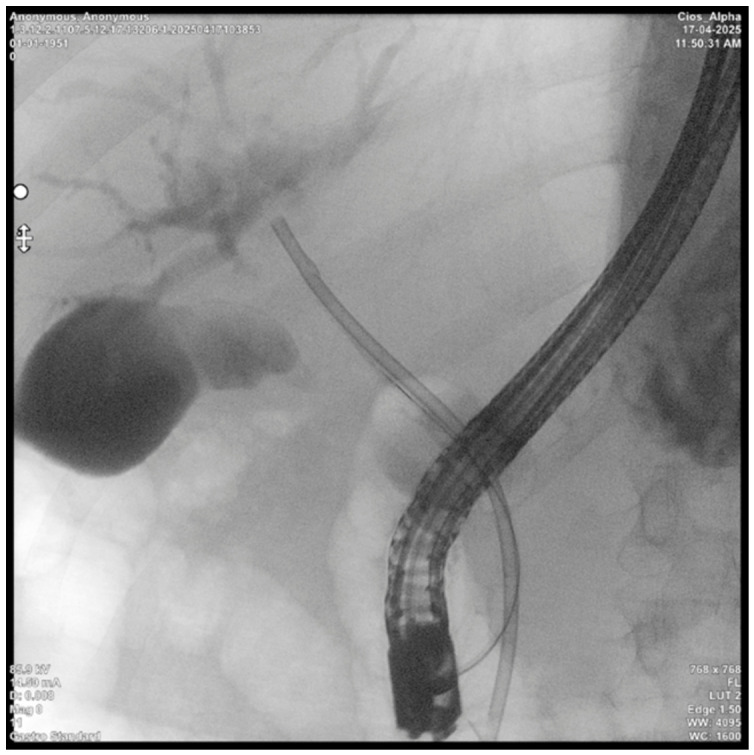
Endoscopic retrograde cholangiopancreatography (ERCP) showing partial stenting of the common bile duct for tumour-related biliary obstruction.

**Figure 16:**
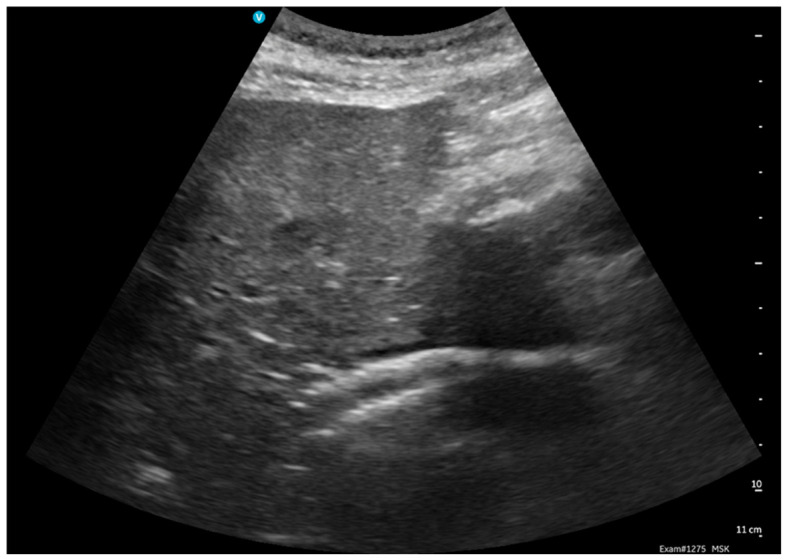
Endoscopic ultrasound-guided hepaticogastrostomy (EUS-HGS), performed to achieve internal biliary drainage after incomplete ERCP drainage.

**Table 1:** Comparative summary of reported cases of renal metastasis from cholangiocarcinoma.

Reference	Age/Sex	CCA Subtype	Prior Treatment	IHC Profile	Outcome
**Choi et al., 2012** [12]	64 y/M	Intrahepatic	Hepatic resection + cholecystectomy	Not reported	Nephrectomy; pulmonary mets; death ~14 mo post-op
**Matsumoto et al., 2020** [11]	72 y/M	Intrahepatic (pT3N0M0)	Segmental hepatic resection	CK7+, CK19+, CK20−, PAX8−	Partial nephrectomy + hepatic resection; alive at 30 mo on GemCis
**Present case, 2025**	72 y/M	Extrahepatic (perihilar/distal)	None—metastasis was initial presentation	CK7+, c-kit weak+, PAX8−	Palliative ERCP + EUS-HGS; ECOG 3 at discharge; GemCis deferred

GemCis: gemcitabine plus cisplatin; EUS-HGS: EUS-guided hepaticogastrostomy.

## Data Availability

The data presented in this case report are not publicly available due to patient privacy and confidentiality considerations. Relevant clinical, imaging, histopathological, and laboratory data supporting the findings of this report are included within the article.

## Abbreviations

The following abbreviations are used in this manuscript:

ALP	Alkaline phosphatase
AUA	American Urological Association
CA 19-9	Carbohydrate antigen 19-9
CAIX	Carbonic anhydrase IX
CCA	Cholangiocarcinoma
CEA	Carcinoembryonic antigen
CEUS	Contrast-enhanced ultrasound
CGP	Comprehensive genomic profiling
CK7	Cytokeratin 7
CK19	Cytokeratin 19
CK20	Cytokeratin 20
CRP	C-reactive protein
CT	Computed tomography
eCCA	Extrahepatic cholangiocarcinoma
ECOG	Eastern Cooperative Oncology Group
EMA	European Medicines Agency
ENT	Ear, nose, and throat
ERBB2	Erb-b2 receptor tyrosine kinase 2
ERCP	Endoscopic retrograde cholangiopancreatography
ESR	Erythrocyte sedimentation rate
EUS	Endoscopic ultrasound
EUS-HGS	Endoscopic ultrasound-guided hepaticogastrostomy
FDA	Food and Drug Administration
FGFR2	Fibroblast growth factor receptor 2
GGT	Gamma-glutamyl transferase
GI	Gastrointestinal
Hb	Haemoglobin
HER2	Human epidermal growth factor receptor 2
iCCA	Intrahepatic cholangiocarcinoma
IDH1	Isocitrate dehydrogenase 1
IHC	Immunohistochemistry
MRI	Magnetic resonance imaging
MUC2	Mucin 2
MUC5AC	Mucin 5AC
PAX8	Paired box gene 8
PET-CT	Positron emission tomography-computed tomography
RCC	Renal cell carcinoma
RCC-Ma	Renal cell carcinoma marker antigen
SEER	Surveillance, Epidemiology, and End Results
